# Sudden unexpected death in Parkinson's disease: Insights from clinical practice

**DOI:** 10.1016/j.clinsp.2021.100001

**Published:** 2022-02-10

**Authors:** Fulvio A. Scorza, Marcia Guimarães-Marques, Mariana Nejm, Antônio Carlos G. de Almeida, Carla A. Scorza, Ana C. Fiorini, Josef Finsterer

**Affiliations:** a Neuroscience Discipline, Escola Paulista de Medicina (EPM), Universidade Federal de São Paulo (UNIFESP), São Paulo, SP, Brazil; b Neuroscience Center of the Woman Health “Professor Geraldo Rodrigues de Lima”, Escola Paulista de Medicina (EPM), Universidade Federal de São Paulo (UNIFESP), São Paulo, SP, Brazil; c Laboratory of Experimental and Computational Neuroscience, Department of Biosystems Engineering, Universidade Federal de São João del-Rei (UFSJ), São João del-Rei, MG, Brazil; d Department of Phonoaudiology, Escola Paulista de Medicina (EPM), Universidade Federal de São Paulo (UNIFESP), São Paulo, SP, Brazil; e Phonoaudiology Post graduation Program, Pontifícia Universidade Católica de São Paulo (PUC-SP), São Paulo, SP, Brazil; f Klinikum Landstrasse, Messerli Institute, Vienna, Austria

**Keywords:** Parkinson's disease, Sudden death, Heart, Prevention

## Abstract

Classified as the second most common neurodegenerative disorder associated with aging after Alzheimer's disease, Parkinson's disease (PD) is the most common movement disorder. In the last decade, despite advances in treatment, mortality rates linked with PD continued to reach significant figures. Available studies have shown that compared with healthy controls, patients with PD are accompanied by high rates of premature death. This is usually caused by factors such as pneumonia and cerebrovascular and cardiovascular diseases. Recently, it has been demonstrated that a significant proportion of patients with PD die suddenly. This is referred to as a sudden and unexpected death in PD (SUDPAR). Here, we focus on the magnitude of SUDPAR. Finally, it is important to learn more about SUDPAR for the implementation of effective prevention strategies.

## Central Message

• To reduce the risk of and prevent sudden unexpected death in Parkinson's disease (SUDPAR) translational research and public campaigns are warranted.
• Patients with Parkinson's disease should be regularly monitored for cardiac, pulmonary, and autonomic disorders.

## Significance statement

In recent years, a considerable number of patients with Parkinson's disease (PD) have been reported to die suddenly. This is known as a sudden and unexpected death in PD (SUDPAR). In this review, we focus on the magnitude of SUDPAR. It is imperative that we study and focus more on SUDPAR for the design and implementation of effective prevention strategies.

## Parkinson's disease: general aspects

The available reports on Parkinson's disease (PD) provide relevant neurological insights on the development and manifestation of the disease.^1^ While the signs of ““shaking palsy”” have been described in approximately 5,000 BC,^2^, ^3^, ^4^ extant literature confirm that PD had been there before the industrial revolution.^4^^,^^5^ In the fifteenth century, Leonardo Da Vinci described a condition similar to that of PD^6^ as “whose soul cannot control their movements in spite of the fact that their extremities are shaking continuously”.^4^^,^^5^^,^^7^^,^^8^ In 1776, at a lecture at the Royal Society, John Hunter described a patient who might have had shaking palsy.^5^^,^^9^^,^^10^ Hunter's observations were not considered by the medical community until 1817, when James Parkinson described the disease.^3^, ^4^, ^5^ His remarks were extraordinarily detailed, making PD the first syndrome to be explained after neurology became a specialty.^3^^,^^4^ Then, the designation of “Parkinson's disease” was further consolidated in 1865 by William Sanders and later diffused by Jean-Martin Charcot, a French neurologist.^11^

Over the past 20 years, there has been great advancement in the field of PD research.^4^^,^^12^ PD is the second most recurrent neurodegenerative disorder associated with aging after Alzheimer's disease and the most common movement disorder.^4^^,^^12^, ^13^, ^14^ It is a complex neurodegenerative disorder characterized by tremor, rigidity, bradykinesia, akinesia, and postural instability due to progressive neuronal loss in the *substantia nigra pars compacta* (SNpc). The clinical syndrome includes motor and non-motor symptoms.^4^^,^^15^, ^16^, ^17^, ^18^ While the clinical features are the basis of PD diagnosis, specific investigations can help differentiate PD from other extrapyramidal disorders.^15^ In industrialized countries, PD is prevalent in 0.3% of the general population, 1.0% in individuals older than 60 years, and 3% in those aged over 80 years, with the incidence rates ranging from 8 to 18 per 100,000 person-years.^4^^,^^15^^,^^16^ PD is estimated to increase to over 50% by 2030, and the only proven risk factor for the onset of the disease is advancing age.^4^^,^^15^^,^^16^^,^^19^^,^^20^

Additionally, sex represents a moderate risk for the development of PD, with males showing a higher chance of developing PD (3:2) than females.^4^^,^^15^^,^^21^^,^^22^ Lifestyle and environmental exposures have been linked to higher (pesticide exposure, prior head injury, rural living, β-blocker usage, agricultural occupation, insufficient water drinking, and stress) or lower (tobacco smoking, coffee drinking, non-steroidal anti-inflammatory drug use, calcium channel blocker use, and alcohol) risk of PD development.^4^^,^^15^^,^^19^^,^^23^

Movement abnormalities in PD can be attributed to the loss of dopaminergic (DA) neurons of the SNpc and widespread intracellular α-synuclein aggregation.^4^^,^^12^^,^^15^^,^^24^ Furthermore, these protein aggregates can be pathological (Lewy bodies) and are often accompanied by dystrophic neurites (Lewy neurites), mostly located in axons.^24^^,^^25^ Interestingly, postmortem analysis of PD brains indicated that loss of approximately 30% of DA neurons in the SNpc causes the onset of motor symptoms.^24^ Additionally, nigral DA neuron loss increases by more than 60% after the onset of motor symptoms, thereby presenting a direct relationship between the severity of motor symptoms and disease duration.^24^ Exquisitely, it was proposed that Lewy body formation and deposition of α-synuclein involves multiple neuronal systems. Thus, this process starts in the glossopharyngeal and vagal nerves, dorsal motor nucleus, and anterior olfactory nucleus, with progressive spread to the brain stem. In the advanced stages, this process spreads to the mesocortex and allocortex, and finally to the neocortex.^15^^,^^25^^,^^26^ The currently available therapies for PD only affect disease symptoms and are predominantly focused on the dopaminergic pathway.^4^^,^^15^^,^^19^ In general terms, the pharmacological striatal DA restitution approach is employed to address both motor and non-motor symptoms as surgical interventions (deep brain stimulation of the subthalamic nuclei) are successfully applied against intractable L-DOPA-related motor complications.^4^^,^^12^

Overall, PD's global burden has more than doubled due to the increasing number of elderly people.^27^ Importantly, there is a rising consensus that cardiovascular disease in patients with PD increases mortality, thereby leading the scientific community to be increasingly alert to these current events.^4^^,^^28^

## Sudden unexpected death in Parkinson disease

### Historical notes on sudden death

Sudden death (SD) has been a part of human history for thousands of years.^29^^,^^30^ In Egypt, 4000 years ago, SD was associated with myocardial infarction.^30^ According to the Ebers papyrus, “when a patient has pain in the arm and left side of the chest, there is a threat of death.^30^”. The first formal sudden cardiac death (SCD) case report was made early in the 4^th^ century BC by the father of medicine, Hippocrates of Kos. He stated in his aphorisms that “those subject to frequent and severe fainting attacks without apparent cause die suddenly”.^31^, ^32^, ^33^ Later in the 14^th^ century, Count Gaston de Foix passed away suddenly after contact with ice water.^30^^,^^34^ He felt chest pain and commented, “I am a dead man. May God have mercy on me”.^30^^,^^34^ In the 18^th^ century, Pope Clement XI ordered Lancisi to write a book evaluating the clinical and pathological data on a series of SD cases.^30^^,^^35^ He described a relationship between SD and the existence of chest pain and the anatomical signs of cardiac disease.^30^^,^^35^ At the end of the same century, Heberden outlined the first description of “angina pectoris”.^30^ In the 19^th^ century, translational studies demonstrated that coronary artery occlusion causes ventricular fibrillation and SD, and that electric shocks can terminate ventricular fibrillation.^36^ In the 20^th^ century, heart disease became the most common cause of death in America.^37^ Today, researchers show great interest in the increased frequency of SD, its association with coronary heart disease, and the importance of ventricular dysfunction in SD.^30^^,^^38^

Although the developed world has overcome the burden of communicable disease-related SD,^39^ SCD has not followed a similar trend, suggesting that the percentage of deaths associated with SCD is still constant or increasing as a percentage of the total mortality.^40^ Along these lines, important information regarding SCD has been described.^41^ Most textbooks define SCD as an “unexpected death occurring within one hour from the onset of symptoms in an individual with stable clinical conditions before the onset of the life-threatening arrhythmic event”.^31^^,^^42^, ^43^, ^44^ Globally, SCD affects more than 7 million lives per year, reflecting the dimension of the problem.^31^^,^^32^ In the USA, the incidence of SCD is between 180,000–400,000 cases per year, depending on its definition.^31^^,^^41^^,^^45^ In Europe, SCD with unsuccessful out-of-hospital cardiopulmonary resuscitation accounts for an average of 350,000 deaths each year.^31^^,^^46^^,^^47^ Specifically, prospective studies performed in the USA, Netherlands, Ireland, and China demonstrated that SCD rates range from 50 to 100 per 100,000 among the general population.^31^^,^^48^ In Brazil, two studies reported similar results. In 2012, Martinelli et al. reported an incidence of 21,270 cases of SCD per year in the metropolitan area of São Paulo.^41^^,^^49^ Three years later, Braggion-Santos et al. described SCD features according to autopsy reports in Ribeirão Preto, Brazil.^41^^,^^50^ Along with the revision of 4501 autopsies, the authors identified 899 cases of SCD (20%), with a rate of 30/100,000 residents/year.^41^^,^^50^ Based on the available scientific data on SD, it is crucial to identify new research areas that could minimize the global burden of SCD and establish effective preventive measures to minimize these tragic events in the general population, including patients with PD. It is important to emphasize that the risk of SCD increases with age, showing a high prevalence among elderly people due to coronary heart disease, systolic dysfunction, and congestive heart failure.^51^

## SD in PD

SD elicits intense surprise, sadness, and questions from family members, caregivers, and friends. In medical literature, the definition of SCD is quite different from that of sudden unexpected death (SUD). SUD refers to death from a cardiac disease within a short period of symptom onset (minutes to hours), often without any alert.^52^, ^53^, ^54^ It is difficult to encapsulate the anguish afflicting the people involved in these tragic events. Any human disease might lead to SUD, but the leading causes are related to cardiovascular and respiratory system diseases and central nervous system (CNS) diseases. Regarding CNS diseases, researchers have not addressed the occurrence of premature deaths in individuals with PD.

Although PD is not a “malignant” disease, a recurrent question about PD continues to persist wherein it is often deliberated if patients could die from PD.^22^^,^^55^ Unfortunately, observational, systematic reviews and meta-analysis data across the years have shown that PD is not a benign condition because it has an increased premature death rate compared with the general population.^4^^,^^14^^,^^17^^,^^56^, ^57^, ^58^, ^59^, ^60^, ^61^, ^62^ Specifically, in the first 5 years after disease onset, mortality in PD does not increase but it increases with a relative risk of 3.5 after 10 years, as evident from the reported data.^4^^,^^12^ In this sense, available literature shows that determinant factors, such as aspiration pneumonia, dementia, advanced age, male sex, cancer, and cardiovascular disease, typically cause death in patients with PD.^16^^,^^22^^,^^56^^,^^60^^,^^62^, ^63^, ^64^, ^65^, ^66^ There is a considerable body of literature concerning cardiac dysfunctions that demonstrates the involvement of the heart in PD, including cardiac autonomic dysfunction, cardiomyopathy, coronary heart disease, and arrhythmias/conduction defects.^4^ Malignant arrhythmias may be triggered by QT prolongation because of the anti-Parkinson medication (alone or in combination) applied. At the same time, the topic of the current debate in the literature is the possible occurrence of SUD in PD (SUDPAR).^4^^,^^14^ Thus, although cases of SUDPAR do not receive sufficient attention from the scientific community, the first reported case dates to the 1970s (Table 1).^4^^,^^14^ In 1976, Rajput and Rozdilsky.^67^ described two instances of SUDPAR in seven individuals with PD as evaluated from their sample. These two patients with PD died suddenly without a toxicological or anatomical explanation for SD as assessed by postmortem autopsy evaluation.^67^ Thirty years later, Sato et al. carefully evaluated the long-term outcomes of 1,768 Japanese patients with PD (793 male and 975 female) over a period of > 10 years.^68^ They found SD in 10 of 131 individuals with PD.^68^ In 2014, Matsumoto et al. assessed the causes of death in patients with PD and verified seven cases of SUDPAR in an autopsy analysis.^69^ In 16 patients with PD who underwent postmortem examinations, they reviewed the clinical data and causes of death. They found that a quarter of these patients had died suddenly, suggesting that a considerable number of patients with PD experienced an SD.^69^ The first long-term follow-up mortality prospective study in patients with PD was recently conducted by Zhang et al..^70^ They provided important evidence on the survival and causes of death in idiopathic PD. They found SD in three out of 31 PD individuals, suggesting that specific interventions adapted to potential risk factors associated with death may provide substantial benefits.^70^ Altogether, 21 patients with SUDPAR have been reported to date.

**Table 1:** Overview of SUDPAR cases reported in the literature.

Clinical Studies	Year of publication	Study design	Number of deaths assessed /number of SUDPAR cases
Rajput and Rozdilsky ^67^	1976	Necropsy	7 deaths/2 SUDPAR = 28.5% of cases
Sato et al. ^68^	2006	Cohort	131 deaths/7 SUDPAR = 5.3% of cases
Matsumoto et al. ^69^	2014	Autopsy	16 deaths/7 SUDPAR = 43.7% of cases
Nishida et al. ^95^	2017	Clinicopathological	2 case reports of SUDPAR
Zhang et al. ^70^	2018	Autopsy	31 deaths/3 SUDPAR = 9% of cases

Although much has been discovered about PD, several aspects of it remain unclear.^16^, and SUDPAR is only a part of this puzzle.^71^^,^^72^ Accordingly, several questions should be raised to elucidate the occurrence of SUDPAR.

**Where?** Being one of the most common neurodegenerative disorders, PD affects at least 10 million people worldwide.^73^ It is associated with a significant burden on disability, comorbidities, stigma, costs, and even mortality.^12^^,^^14^ Thus, neuroscientists do not doubt that patients with PD have a reduced life expectancy due to increased mortality compared to the general population.^4^^,^^12^^,^^14^ Although SUDPAR is still considered a rare phenomenon, it is a significant cause of death in patients with PD.^4^^,^^14^^,^^71^^,^^72^

**What?** The gap in autopsy studies and the rarely witnessed or monitored cases of SUDPAR complicate an accurate discussion regarding its definition. Didactically, SUDPAR has been defined as SUD in a patient with PD without any satisfactory explanation of death, as determined by autopsy studies.^4^^,^^14^^,^^71^^,^^72^

**When?** There has been a dearth of research centers for movement disorders that have promoted accurate studies demonstrating the possible incidence of SUDPAR. This lack of data is probably due to patient population differences, study design, and incomplete documentation. However, relevant global analysis of SUDPAR has demonstrated that 14% of patients with PD die suddenly.^4^

**Why?** Recent research suggests that multiple clinical risk factors might contribute to SUDPAR. The most common risk factors of SUDPAR are age at onset, duration of PD, sex, motor severity, concomitant cardiac and pulmonary disease, drug treatment (polypharmacy), and sleep disorders/circadian alterations.^4^^,^^14^^,^^72^

**How?** Comprehensive explanations on the underlying mechanisms of SUDPAR have been reported,.^4^^,^^14^^,^^72^ and it is extremely important to understand them as this would lead to the identification of previously unrecognized risk factors.^4^^,^^14^^,^^72^ The most recent data show that cardiac abnormalities and autonomic dysfunction possibly play “direct” role in SUDPAR because approximately 60% of patients with PD have cardiovascular disturbances and because of the frequent autonomic disorders in PD.^4^^,^^14^^,^^72^

Overall, cardiac abnormalities and autonomic dysfunction are the major causes of SUDPAR.^4^ Therefore, prevention remains the best approach in these circumstances.

## Preventive measures and SUDPAR

While SUDPAR represents a severe outcome of PD, there exists no evidence-based prevention against SUDPAR. A fundamental practical problem in studying risk factors associated with SUDPAR, incidence mechanisms, and prevention is that it is relatively unusual. Thus, to avoid premature mortality, it would be interesting to develop preventive studies in patients with PD to evaluate risk factors.^71^^,^^72^ In this sense, the strong epidemiologic and pathophysiologic link between PD and SUDPAR suggests that efforts must be made to minimize the risk of this tragic event. Therefore, in the given context, the following general recommendations can be provided:

**Education of patients, family members, and caregivers:** A consensus exists that there is an educational problem with SUDPAR. It is important to disseminate and improve education in SUDPAR, including reinforcing adherence to treatment by patients. Educating people is challenging and complicated, but it is a way to supply access to human emancipation and social transformation.^74^ One crucial question is to determine the correct time to talk about SUDPAR to individuals, family members, and caregivers. It would be appropriate to establish a task force that discusses issues related to SUDPAR to answer these questions.
**The convergence of clinical research and clinical care:** Physicians, including neurologists and cardiologists, and other multidisciplinary team members should develop close collaboration to provide guidance for how to assess the risk of SUDPAR and how to manage it. This multidisciplinary team should perform routine cardiovascular screening (electrocardiogram, Holter monitoring, and echocardiography) to ensure a steady decrease in the mortality rates of individuals with PD.
**The importance of staying hydrated for patients with PD:** Water is crucial for life and is one of the six essential nutrients together with carbohydrates, fats, vitamins, proteins, and minerals. Water is frequently consumed as a dietary constituent.^75^, ^76^, ^77^, ^78^ The recommendation for sedentary adults is to drink approximately 2 L daily.^75^^,^^78^^,^^79^ Furthermore, age-related water consumption changes make elder adults vulnerable to dehydration, which is considered the most common fluid and electrolyte disorder in older adults.^75^^,^^80^^,^^81^ Dehydration is a risk factor for clinical deterioration in patients with PD, as individuals with dysphagia drink only about 1 L of water daily. Thus, it is strongly recommended that patients with PD drink enough water to maintain appropriate hydration^75^^,^^82^, ^83^, ^84^ and that adequate hydration level is part of clinical practice guidelines for PD and future research on SUDPAR.^75^
**Benefits of fish consumption for patients with PD:** Recent preclinical and clinical studies have proposed that omega-3 polyunsaturated fatty acids (n-3 PUFAs) could be beneficial for patients with PD because of their anti-inflammatory and metabolic properties.^85^^,^^86^ Since the human body cannot provide n-3 PUFAs, the primary sources of these essential fatty acids are nutrients rich in fish and other seafood, mainly anchovies, Atlantic herring, salmon, trout, and sardines.^87^, ^88^, ^89^, ^90^, ^91^, ^92^ Furthermore, people who crave a diet free of chemical pollutants and wish to obtain the advantages of n-3 PUFAs, can opt for fish oil supplements or foods such as nuts or oils (linen, canola, and soy).^87^, ^88^, ^89^, ^90^, ^91^, ^92^ Several studies recently found that n-3 PUFAs protect against cardiovascular morbidity and mortality and can successfully avert sudden unexpected death in epilepsy (SUDEP),^93^ it is prudent to assume that n-3 PUFAs have a beneficial effect on SUDPAR. Although innovative, this proposal for encouraging the consumption of n-3 PUFAs against SUDPAR is still speculative, and researchers must conduct translational studies to establish its effectiveness with precision. However, eating fish is healthy, inexpensive, and unlikely to produce any side effects.
**Management of orthostatic hypotension in patients with PD:** Autonomic dysfunction is very common in PD.^4^^,^^94^^,^^95^ Approximately 71% of patients with PD have one or more autonomic dysfunction manifestations that severely affect their quality of life.^94^^,^^96^ In fact, orthostatic hypotension (OH) is an autonomic dysfunction associated with PD that mostly debilitates patients, having a deep impact on a patient's quality of life, as it promotes an increase in morbidity and mortality index.^97^ As approximately 50% of patients with PD have OH, treatment is essential.^97^ Measures that have been implemented in the management of OH include correction of aggravating factors, implementation of non-pharmacological measures, and pharmacologic therapies, including fludrocortisone, midodrine, droxidopa, and norepinephrine reuptake inhibitors.^97^

## Final considerations

PD is a systemic neurodegenerative disorder that is heterogeneous, multifactorial, and merits further research. The process of obtaining a comprehensive understanding of the disease is quite long. First, the family history of the patients with PD, particularly the occurrence of sudden premature death, should be evaluated to detect any possible cardiac syndrome. Second, to reduce heart diseases and possible tragic events, patients with PD should adopt additional preventive behaviors, such as abstinence from smoking, control of alcohol intake, exercise, weight reduction, control of high blood pressure, blood glucose, and lipid dysfunction. Third, to better understand the epidemiology, pathophysiological basis, potential risk factors, and preventive measures of SUDPAR, well-designed clinical studies and basic research in relevant animal models should be undertaken. Fourth, PD must receive closer attention from governments, policymakers, authorities, and healthcare professionals across all continents. Translational research on mortality and other aspects of PD is crucial to improve our understanding and ultimately improve the quality of life of PF patients.^71^^,^^72^ Finally, we concur with the proposal.^98^ that public campaigns that contributed to a marked reduction in the incidence of sudden infant death syndrome could be used as an inspiration and incentive in the SUDPAR field.

## Author contributions

Scorza FA conceived the presented idea and contributed toward writing the final manuscript. Guimarães-Marques M, Nejm M, de Almeida ACG, Scorza CA, Fiorini AC and Finsterer F contributed toward writing the final manuscript.

## Conflicts of interest

The authors declare no conflicts of interest.
